# Diagnosis of Some of the Conditions That Affect Breast-Fed Babies

**Published:** 1917-06

**Authors:** B. E. Greer

**Affiliations:** Dallas, Texas


### DIAGNOSIS OF SOME OF THE CONDITIONS THAT AFFECT BREAST-FED BABIES.

BY DR. B. E. GREER, DALLAS, TEXAS.

Infants reared on mothers' milk are almost invariably healthier, stronger and less troublesome than those that are bottle-fed, less susceptible to disease, and show a greater resistance to disease, in so much that the mortality rate is about one-sixth that of the bottle-fed. Thirty-one per cent of the infant mortality among the bottle-fed are fed the bottle by their mothers, 50 per cent are fed by strangers, the remainder fed by both. Showing the importance of mothers feeding their own babies, even if it is an artificial food.

On account of the higher mortality rate among the bottle-fed, if for no other reasons (and there are other reasons), every healthful mother should endeavor to nurse her offspring wholly or partially, even if it be only for a brief period of time. We can relieve the mother of a great deal of the drudgery of nursing by giving the baby one or two bottles a day of some artificial food, preferably cow's milk as the basis, and by showing the mother this consideration she will be more willing to nurse her baby, and we will hear less complaint about her health or being unable to properly nurse her baby. Nordheim collected statistics from a large number of mothers who did not nurse their offspring, and found that 86.7 per cent had no good reason for not nursing, except selfish motives. I do not want to be misunderstood that I am advocating that there are no conditions under which a mother should not nurse her baby. There are diseased conditions under which she should not, but this paper is not intended to deal with diseased conditions, but overlooked conditions, where disease is not a part.

Very few mothers' milk will be normal under ten days or two weeks after delivery, especially if they have had unnatural conditions at that time to contend with.

During menstruation, the proteids are markedly increased, and the fats are markedly decreased in the mother's milk. This sometimes causes an indigestion in the baby, but usually of only a day or two duration, and consequently one is not justified in weaning the baby.

The amount of exercise and the diet of the mother should be governed by her physical condition, and her digestive capacity, and the quantity and quality of her milk.

The next problem is healthful surroundings, hygiene of mother and baby, manner in which both are clothed with reference to the time of the year, with cheerful surroundings and freedom from anxiety and care.

A mother should never nurse her baby when she is very tired, hot, or in an angry state. The wild animals of the forest will not permit of their young nursing immediately after a fight, but will hide away for hours, to prevent their nursing, as they have learned that nursing at that time will make their offspring sick, yet many a human mother will disregard these simple precautions.

Permitting a baby to nurse too rapidly, too frequently, in too large amount, roughly handling them immediately after feeding, not giving a sufficient amount of water, giving of unnatural food for a child of that age, etc.; all are sources of bowel disturbances in the young.

